# Investigation of the psychometric properties of the Comprehensive Feeding Practices Questionnaire in Turkish parents

**DOI:** 10.1017/S1368980024001125

**Published:** 2024-05-30

**Authors:** Ceren Şarahman-Kahraman, Cansu Memiç-İnan, Nurcan Yabanci-Ayhan, Ayse Özfer Ozcelik

**Affiliations:** 1 Department of Nutrition and Dietetics, University of Alanya Alaaddin Keykubat, Alanya/Antalya, Turkey; 2 Department of Nutrition and Dietetics, Niğde Ömer Halisdemir University, Niğde, Turkey; 3 Department of Nutrition and Dietetics, University of Ankara, Ankara, Turkey

**Keywords:** Child nutrition, Feeding practices, Children’s eating behaviours, Parent-child relations, Children’s eating scale

## Abstract

**Objective:**

The Comprehensive Feeding Practices Questionnaire (CFPQ) measures parental attitudes towards feeding practices that directly influence children’s eating habits. This study aims to determine the reliability and validity of the Turkish adaptation of the CFPQ developed by Musher-Eizenman *et al.*

**Design:**

Validity and reliability analyses were conducted for the Turkish version of the CFPQ (T-CFPQ). In addition to reliability analyses and partial correlations between scale dimensions, correlations between scale dimensions according to mothers’ BMI and children’s BMI *z*-scores were also examined.

**Setting:**

Parents with children aged 18 months to 8 years living in the community.

**Participants:**

The study sample consisted of 274 parents with children aged 18 months to 8 years who agreed to participate in the online survey.

**Results:**

In this study, forty-seven items and twelve-factor structure describing feeding practices were supported by the confirmatory factor analysis. Although most of the dimensions of the T-CFPQ showed significant correlations with each other, the highest correlation was found between the encourage balance/variety and the dimension of modelling and teaching nutrition (*r* = 0·53; 0·50) (*P* < 0·05). There was a negative correlation between the child’s BMI *z*-score and the pressure to eat dimension (*r* = –0·173; *P* < 0·01) and a positive correlation between the restriction for weight dimension (*r* = 0·339; *P* < 0·01). Maternal BMI was negatively associated with the involvement dimension (*r* = –0·121; *P* < 0·05) and positively associated with the restriction for weight dimension (*r* = 0·154; *P* < 0·01).

**Conclusions:**

The findings revealed that the T-CFPQ is a valid and reliable measurement tool that can be applied to obtain the necessary information for evaluating nutritional interactions between parent and child.

Childhood obesity is becoming a serious public health problem. According to the Monitoring of Growth in School Age Children in Turkey (TOÇBI-2011) Project Survey^(1)^, 14·3 % of children aged 3–6 years were overweight, and 6·5 % were obese. Similarly, the Childhood Obesity Survey in Turkey conducted in 2016^(2)^ found that 14·6 % of children aged 6–10 years were overweight, and 9·9 % were obese. As with the rest of the world, the prevalence of childhood obesity has increased in Turkey in recent years. It is estimated that if the current rate of increase continues, the number of overweight and obese children worldwide may reach 70 million in 2025^(3)^. The aetiology of obesity is influenced by both environmental and genetic factors. Eating behaviours are reported to be one of the most important environmental factors affecting obesity, and it has been reported that the foundation of most of the eating behaviours is laid in childhood^(4)^.

Children receive their initial nutrition education from their parents, who have a direct impact on the development of eating habits during the preschool years^(4)^. Parents influence their children’s eating behaviours by promoting the consumption of healthy foods and restricting or limiting the intake of foods that are detrimental to health^(5,6)^. Furthermore, various physical, social and emotional environments influence parents’ feeding practices and, as a result, their children’s eating behaviours^(7)^. The purposeful behaviours and guidelines that parents use to influence which foods, when and in what amounts their children consume are called parental feeding practices^(8)^. These practices have a direct impact on children’s body weight. Restrictive and controlling parental feeding practices are generally associated with higher body weight, whereas eating pressure applied to children is associated with lower body weight^(9)^. As childhood obesity can have long-term effects on health in adulthood, it is important to target early feeding experiences, such as parental feeding practices, for successful interventions^(10)^.

Assessing the underlying causes of parental attitudes and behaviours can be challenging due to their abstract and complex nature^(11)^. In addition, the lack of validated measurement tools assessing parental feeding behaviours and styles has made it difficult to compare research on this topic^(12)^. Upon reviewing the literature, it becomes apparent that most parental feeding practices are limited to a few practices, such as restrictive feeding and pressure to eat. These practices aim to control a child’s food intake and are often evaluated using the Child Feeding Questionnaire. The Child Feeding Questionnaire is a thirty-one-item self-report questionnaire that measures three dimensions of parental feeding practices: restriction, pressure to eat, and monitoring^(13)^. Although controlled feeding practices implemented by parents aim to ensure balanced nutrition in children, children of parents who are overly controlling on food consumption consume foods with high-fat content and high amounts of snacks^(5,14)^. Frequent emphasis on parental control for feeding practices may cause other feeding practices to be ignored. Therefore, parental modelling to create a healthy food environment is another effective nutrition practice. It has been reported that parents’ orientation towards teaching healthy nutrition to their children is a point that is not examined in parental feeding practices^(15)^.

The Comprehensive Feeding Practices Questionnaire (CFPQ)^(15)^ is a well-developed scale that addresses feeding practices broadly. It has a systematic approach and includes concepts that promote child health, such as modelling healthy eating and creating a healthy food environment beyond the control of feeding practices^(16)^. The scale consists of twelve factors and forty-nine items. It was developed based on three studies that evaluated the comprehensive nutritional behaviours of parents with young children aged between 18 months and 8 years. In the first study conducted with American parents of children aged 2–8 years, the scale showed reasonable validity and reliability^(15)^. The CFPQ has been validated in countries other than the USA, such as Jordan, New Zealand, Brazil and Malaysia^(17–20)^. When evaluating the psychometric properties of the CFPQ in French parents with children aged 4–7 years, nine factors demonstrated reasonable validity and reliability^(21)^. Haszard *et al.*^(18)^ evaluated the CFPQ in a large sample of 1013 children aged 4–8 years in New Zealand and reported that the original twelve-factor structure was not appropriate and confirmed the five-factor model consisting of healthy eating guidance, monitoring, parent pressure, restriction and child control. Our study aimed to determine the reliability and validity of the CFPQ to the Turkish culture.

## Methods

### Participants

The sample of the research was planned to consist of parents who live in Turkey, have children between the ages of 18 months and 8 years and volunteered to participate in the research. In the adaptation of a scale to a different language and culture, the sample size recommended to determine its validity and reliability should be at least 5–10 times the number of items in the scale^(22)^. Since the number of items on the scale was 49, the study was planned to be conducted with a minimum of 245 parents. In this study, the snowball sampling method was used to reach as many parents as possible with children between the ages of 18 months and 8 years. The study was explained in detail to the first person who was thought to represent the target group of the study. Before sending the questionnaires to people who were similar to the first person and who were recommended by the first person, these people were contacted by phone or email and checked whether they were suitable for the study. The questionnaires were delivered online to the eligible people. With the suggestion of each sampling unit interviewed, the other sampling unit was also contacted and checked in the same way. In this way, it was ensured that the data were homogenous, the measurement tool adequately represented the population, its internal consistency increased, and the results were reliable.

### Adaptation protocol

To adapt the scale into Turkish, permission was obtained via email from Musher-Eizenman *et al.*^(15)^, the creators of the scale, for the use and translation of the scale. For the adaptation of the CFPQ into Turkish, the original version of the forty-nine-item scale was first translated into Turkish. The translation of the Turkish version of the CFPQ (T-CFPQ) was evaluated by three experts with a good command of English using the translation-back-translation method^(23)^. The consistency and semantic integrity of the translated forms were evaluated by ten experts in the field of nutrition and dietetics, and the adaptation process of the scale was finalised after the necessary corrections were made. In addition, an online pilot study was conducted on thirty mothers to determine the comprehensibility of the items in the scale. The data collected in the pilot study were not included in the analysis of this study.

## Measures

### Questionnaire form

In the first part of the questionnaire, information about the demographic characteristics such as age, sex, height and body weight of the children and their mothers was collected. Height (cm) and body weight (kg) data of children and mothers were obtained based on the mothers’ declaration. The second part of the questionnaire includes T-CFPQ items.

### Comprehensive Feeding Practices Questionnaire (CFPQ)

It is a scale developed by Musher-Eizenman *et al.* to measure parents’ attitudes towards comprehensive feeding practices related to their children’s nutrition^(15)^. In this study, the T-CFPQ consisting of forty-nine items and twelve factors was used. These factors are monitoring, emotion regulation, food as a reward, pressure to eat, child control, teaching nutrition, healthy environment, restriction for weight, restriction for health, modelling, involvement and encourage balance and variety (see Table 1). The scale was scored on a five-point Likert scale from 1 to 5 as ‘never, rarely, sometimes, mostly, always’ for items 1–13 and from 1 to 5 as ‘disagree, slightly disagree, neutral, slightly agree, agree’ for items 14–49. The reverse-coded items are 16 and 37 (see online Supplementary Material).

**Table 1:** Factors of the Turkish version of the Comprehensive Feeding Practices Questionnaire, abbreviations and descriptions

Factor name	Abbreviation	Description
Child control	CC	Parents’ control of the child’s eating behaviour
Emotion regulation	ER	Parents’ use of food to regulate the child’s mood
Encourage balance/variety	EB	Parents’ promoting a balanced diet
Healthy environment	HE	Healthy foods available at home
Food as a reward	FR	Parents’ use of food as a reward for the child’s behaviour
Involvement	IN	Encouraging the child’s participation in meal planning and preparation
Modelling	MD	Parents’ actively demonstrating healthy eating behaviours for their children
Monitoring	MN	Parents’ monitoring of their children’s consumption of less healthy foods
Pressure to eat	PE	Parents’ pressure on the child to consume more food at meals
Restriction for health	RH	Restricting the child’s food intake to limit the consumption of less healthy foods or sweets
Restriction for weight	RW	Restricting the child’s food intake to reduce or maintain body weight
Teaching nutrition	TN	Parents’ use of explicit and instructive techniques to encourage their children’s consumption of healthy foods

### Data analysis

BMI for mothers was calculated as body weight divided by the square of height (kg/m^2^). According to the WHO classification, the mother’s BMI was classified as underweight for <18·5 kg/m^2^, normal for 18·5–24·99 kg/m^2^, overweight for 25·0–29·99 kg/m^2^ and obese for ≥30 kg/m^2(24)^. The BMI *z*-scores of children by age were evaluated using the ‘WHO Anthro’ programme for children aged 0–5 years and the ‘WHO Anthro Plus’ programme for children over 5 years^(25)^. The BMI *z*-scores of children by age were evaluated as follows: <–3 *z*-score as extremely underweight, ≥–3 and <–2 *z*-score as underweight, ≥–2 and <+1 *z*-score as normal, ≥1 and <+2 *z*-score as overweight, ≥+2 and <+3 *z*-score as obese and ≥+3 *z*-score as extremely obese^(26)^.

Descriptive statistics, such as percentages, frequencies, means and sd, were used to determine the characteristics of the participants. The software programmes SPSS 22·0 and AMOS were used. Confirmatory factor analysis (CFA) was performed to confirm the twelve-dimensional structure of the T-CFPQ in Turkish culture. Before the CFA, the data set was checked for incorrect data entry and missing data, and there were no instances of missing data and incorrect data entry. To decide on the appropriate estimation method, the assumption of multiple normal distribution was examined. When Mardia’s test, which is a statistic based on kurtosis and skewness functions, is less than 3, the assumption of multivariate normality is met^(27)^. The Mardia statistic was calculated to determine the multiple normal distribution, and the Mardia statistic value was found 262·417. Since the Mardia statistic was greater than 3, it was concluded that the data did not show multiple normal distribution, and the robust maximum likelihood method was used as the estimation method. In addition, four items (7, 8, 9, 45) showed floor effect, and nineteen items (1, 2, 3, 4, 13, 15, 18, 20, 21, 24, 25, 26, 28, 38, 43, 44, 46, 47, 48) showed ceiling effect. Internal consistency analysis was conducted using Cronbach’s alpha and McDonald’s ω coefficient to determine the reliability of the scores obtained from the Comprehensive Nutrition Practices Scale^(28)^. The Kaiser-Meyer-Olkin test was used to test whether the data were suitable for factor analysis. A Kaiser-Meyer-Olkin value of >0·50 indicates that sampling adequacy of the relevant scale data is suitable. Bartlett’s sphericity test was used to determine whether the correlation matrix was an identity matrix or not. If the *P*-value of Bartlett’s sphericity test is less than 0·05, it means that it was not an identity matrix and there was a correlation between the items. In addition, to determine the relationship between the T-CFPQ subscales, a partial correlation was performed by controlling for the child’s sex, age and BMI *z*-score. The relationship between T-CFPQ subscales and children’s BMI *z*-scores and mothers’ BMI was analysed by Spearman’s correlation test. *P* < 0·05 was accepted as the statistical significance level in all analyses.

## Results

The study involved 274 mothers with children aged between 2 and 8 years. Table 2 presents the characteristics of the participants in the study group.

**Table 2:** Demographic and anthropometric characteristics of children (*n* 274)

Demographic and anthropometric characteristics	Category	*n*	%
Child sex	Male	145	52·92
	Female	129	47·08
Child’s BMI *z*-score	Extremely underweight (<–3)	12	4·4
	Underweight (≥ –3 and < –2)	10	3·6
	Normal weight (≥ –2 and < +1)	143	52·2
	Overweight (≥ +1 and < +2)	59	21·5
	Obese (≥ +2 and < +3)	42	15·3
	Extremely obese (≥ +3)	8	2·9
Maternal education	Primary school graduate	26	9·49
	High school graduate	64	23·36
	Bachelor’s degree	148	54·01
	Postgraduate	36	13·14
Maternal occupation	Public officers	103	37·59
	Worker	47	17·15
	Self-employment	34	12·41
	Housewife	77	28·10
	Not working/unemployed	13	4·75
Family’s income	Low	22	8·03
	Middle	235	85·77
	High	17	6·20
Maternal BMI	Underweight	6	2·2
	Normal weight	140	51·1
	Overweight	86	31·4
	Obese	42	15·3
Worried about the child’s body weight	No worries	207	75·55
	Worried about weakness	46	16·79
	Worried about fatness	21	7·66
Thinking you are responsible for your child’s eating habits	Yes	260	94·89
	No	14	5·11
	Min.–max.	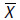	sd
Child’s age (year)	2·00–8·00	5·58	1·64
Child’s BMI (kg/m^2^)	7·30–37·30	17·52	4·28
Mother’s age (year)	23·00–53·00	35·10	4·97
Mother’s BMI (kg/m^2^)	17·60–54·20	25·64	4·73

When Table 2 is examined, 52·92 % of the mothers in the study group had male children, and 47·08 % had female children. Of the mothers, 54·01 % had bachelor’s degrees, 23·36 % were high school graduates, 37·59 % were public officers, and 28·10 % were housewives. In addition, 85·77 % of the families of the children had a middle-income level. 75·55 % of the mothers did not worry about their child’s body weight, while 94·89 % believed that they were responsible for their child’s eating habits. The children had a mean age of 5·58 ± 1·64 years, with 52·2 % having a normal BMI *z*-score and a mean BMI of 17·52 ± 4·28. The mean age of the mothers was 35·10 ± 4·97 years, 51·1 % had a normal BMI, and the mean BMI was 25·64 ± 4·73. Accordingly, the result of the Kaiser-Meyer-Olkin test statistics was 0·807, and it showed that the data was suitable for factor analysis. The results of Bartlett’s test of sphericity (χ^2^ = 4968·838; *P* < 0·0001) indicated that the correlation matrix was significantly different from an identity matrix and there was a correlation between the items.

CFA was conducted to verify the twelve-dimensional structure of the T-CFPQ. As a result of the CFA, the standardised factor loading value of item 42 under the dimension of education about nutrition was –0·04, and the error variance was 1·00. When the correlation of an item with a factor is 0·30 and above, it shows that the item is effective in explaining that factor, and when the factor loading value is lower than 0·30 but the error variance is higher than 0·90, it shows that the item does not serve the dimension^(29)^. As a result, item 42 was excluded from the analysis, and the process was repeated. After repeated analysis, the standardised factor loading value of item 18 under the restriction for weight dimension was 0·28, and the error variance was 0·92; this item was excluded from the analysis, and the analysis was repeated. As a result of the CFA conducted after the two items were removed from the analysis, the factor loadings for all items were higher than 0·30. Table 3 presents the obtained factor loadings and significance values.

**Table 3:** Confirmatory factor analysis results of the Turkish version of the Comprehensive Feeding Practices Questionnaire

Item’s no	Factor load	Error variance	*t*	*P*	Item’s no	Factor load	Error variance	*t*	*P*
Monitoring					Restriction for health				
1	0·78	0·39	–	–	21	0·41	0·83	–	–
2	0·84	0·30	11·59	< 0·05	28	0·59	0·65	6·01	< 0·05
3	0·76	0·42	8·86	< 0·05	40	0·58	0·66	5·56	< 0·05
4	0·80	0·36	8·84	< 0·05	43	0·64	0·59	5·50	< 0·05
Emotion regulation					Teaching nutrition				
7	0·46	0·79	–	–	25	0·86	0·25	–	–
8	0·73	0·47	5·56	< 0·05	31	0·54	0·71	9·34	< 0·05
9	0·93	0·14	5·48	< 0·05					
Food as a reward					Encourage balance/variety				
19	0·78	0·39	–	–	13	0·47	0·78	–	–
23	0·70	0·51	7·89	< 0·05	24	0·46	0·79	4·07	< 0·05
36	0·45	0·80	5·96	< 0·05	26	0·66	0·57	5·65	< 0·05
					38	0·50	0·75	3·89	< 0·05
Child control					Pressure to eat				
5	0·42	0·83	–	–	17	0·37	0·86	–	–
6	0·55	0·70	4·71	< 0·05	30	0·68	0·54	4·62	< 0·05
10	0·59	0·65	4·94	< 0·05	39	0·69	0·52	4·83	< 0·05
11	0·57	0·68	5·32	< 0·05	49	0·52	0·72	4·20	< 0·05
12	0·35	0·88	3·69	< 0·05					
Modelling					Healthy environment				
44	0·61	0·63	–	–	14	0·46	0·79	–	–
46	0·70	0·51	6·83	< 0·05	16	0·56	0·68	5·00	< 0·05
47	0·82	0·33	7·94	< 0·05	22	0·46	0·79	5·10	< 0·05
48	0·81	0·35	8·37	< 0·05	37	0·57	0·68	5·42	< 0·05
Restriction for weight					Involvement				
27	0·67	0·55	–	–	15	0·58	0·67	–	–
29	0·60	0·64	9·52	< 0·05	20	0·54	0·71	5·78	< 0·05
33	0·69	0·53	11·06	< 0·05	32	0·58	0·66	6·56	< 0·05
34	0·73	0·47	11·42	< 0·05					
35	0·68	0·54	10·96	< 0·05					
41	0·58	0·66	8·62	< 0·05					
45	0·41	0·83	5·07	< 0·05					

**Fig. 1:**
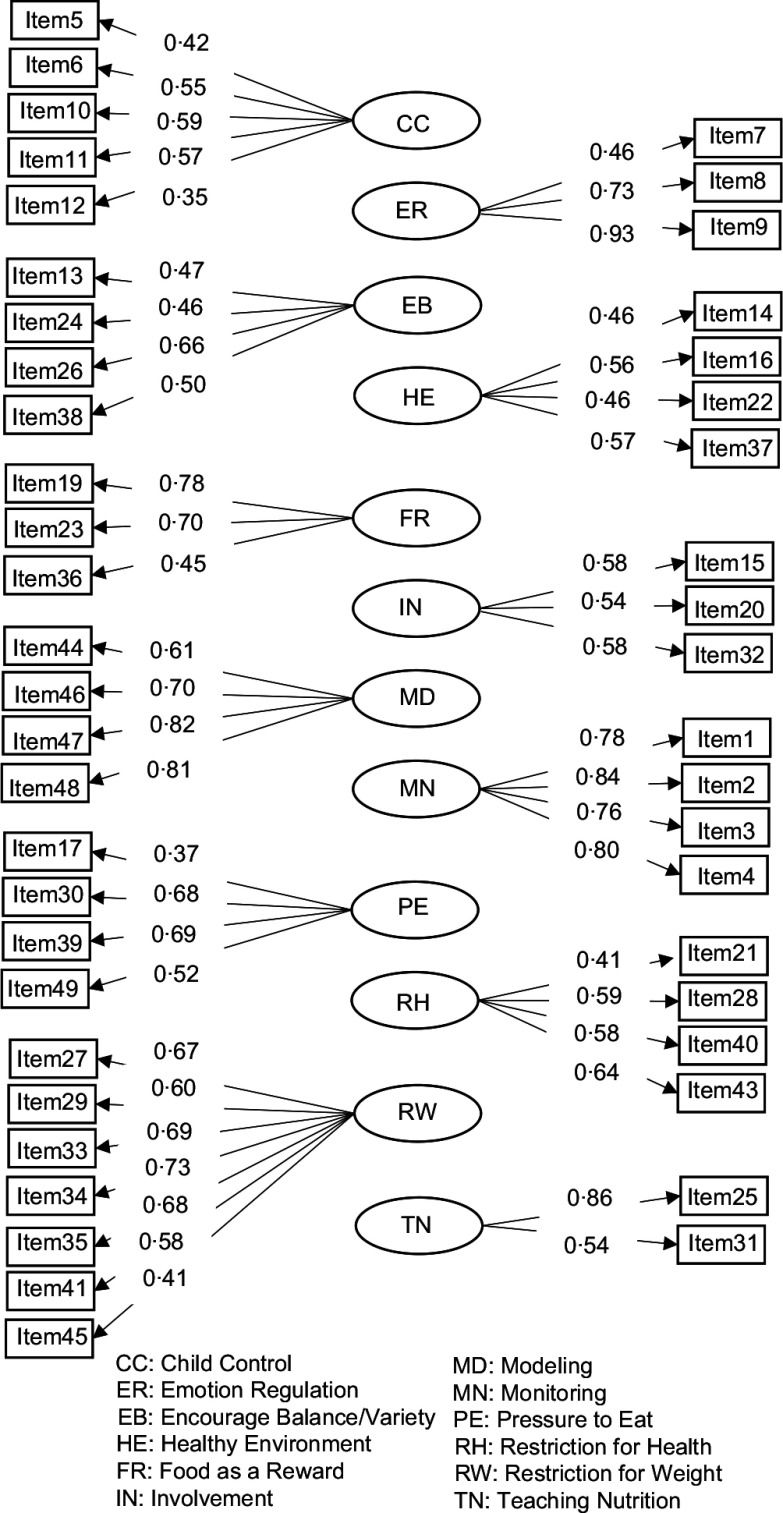
Confirmatory factor analysis of the Comprehensive Feeding Practices Questionnaire (T-CFPQ) Measurement Model

When Table 3 is examined, all items have factor loading values greater than 0·30 and error variances of 0·90 or less. Furthermore, all items were found to be statistically significant (*P* < 0·05). Therefore, it can be inferred that the items effectively measure the construct in their respective factors. The fit index values obtained as a result of CFA are given in Table 4. In addition, the measurement model obtained as a result of the analysis is given in Fig. 1.

**Table 4:** Turkish version of the Comprehensive Feeding Practices Questionnaire confirmatory factor analysis fit index results

	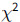	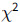 /sd	*P*	CFI	IFI	TLI	RMSEA
Scale	1496·11	1·55	0·000	0·94	0·94	0·93	0·045
Recommended		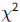 /sd ≤ 3		≥ 90	≥ 90	≥ 90	≤ 0·080

CFI, Comparative Fit Index; IFI, Incremental Fit Index; TLI, Tucker–Lewis Index; RMSEA, Root Mean Squared Error of Approximation.

When Table 4 is examined, the χ^2^/*sd* value is less than 3, indicating a good fit of the model to the data. The Comparative Fit Index value is 0·94, the Incremental Fit Index value is 0·94, and the Tucker–Lewis Index value is 0·93, and since these values are above 0·90, the model fits the data well. When evaluated in terms of the Root Mean Squared Error of Approximation (RMSEA) index, this index was 0·045 for the model, and according to this index, the model is compatible with the data. When evaluating the fit indices overall, the twelve-dimensional model is the best fit for the data. As seen in Fig. 1, items 2, 4, 9, 25, 47 and 48 have the highest factor loadings, while items 5, 12, 17, 21 and 45 have the lowest factor loadings. To determine the reliability of the T-CFPQ scores, Cronbach’s alpha and McDonald’s ω coefficient were calculated as internal consistency analysis. The results are presented in Table 5.

**Table 5:** Reliability coefficient values of the Turkish version of the Comprehensive Feeding Practices Questionnaire subscales

	Item’s no	Cronbach’s alpha	McDonald’s ω
Child control	5	0·61	0·62
Emotion regulation	3	0·73	0·76
Encourage balance/variety	4	0·60	0·61
Healthy environment	4	0·58	0·59
Food as a reward	3	0·64	0·68
Involvement	3	0·59	0·59
Modelling	4	0·81	0·83
Monitoring	4	0·87	0·87
Pressure to eat	4	0·64	0·66
Restriction for health	4	0·65	0·66
Restriction for weight	7	0·82	0·82
Teaching nutrition	2	0·59	0·64

When Table 5 is examined, the Cronbach’s alpha values for T-CFPQ subscale scores range from 0·58 to 0·87, while the McDonald’s ω values range from 0·59 to 0·87. For reliability measurements, values below 0·50 are considered low reliability, values between 0·50 and 0·80 are considered moderately reliable, and values above 0·80 are considered highly reliable^(30)^. Therefore, it can be concluded that T-CFPQ scores are reliable.

After controlling for the sex, age and BMI *z*-score of the children, a partial correlation analysis was conducted to determine the relationship between the T-CFPQ subscales. The results are given in Table 6.

**Table 6:** Associations between Turkish version of the Comprehensive Feeding Practices Questionnaire subscales after controlling for child’s sex, age and BMI *z*-score

	1	2	3	4	5	6	7	8	9	10	11	12
Child control (1)	1·00											
Emotion regulation (2)	0·20^**^	1·00										
Encourage balance/var. (3)	0·04	− 0·21^**^	1·00									
Healthy environment (4)	0·21^**^	0·00	0·20^**^	1·00								
Food as a reward (5)	0·21^**^	0·23^**^	− 0·04	0·36^**^	1·00							
Involvement (6)	− 0·04	− 0·11	0·38^**^	0·07	− 0·05	1·00						
Modelling (7)	− 0·01	− 0·11	0·53^**^	0·12	0·07	0·31^**^	1·00					
Monitoring (8)	− 0·12^*^	− 0·19^**^	0·39^**^	0·01	− 0·08	0·34^**^	0·22^**^	1·00				
Pressure to eat (9)	0·01	0·21^**^	0·00	0·20^**^	0·38^**^	− 0·11	0·07	− 0·02	1·00			
Restriction for health (10)	− 0·08	− 0·14^*^	0·30^**^	0·09	0·09	0·16^**^	0·39^**^	0·27^**^	0·09	1·00		
Restriction for weight (11)	− 0·06	− 0·05	0·12	0·24^**^	0·27^**^	0·11	0·14^*^	0·06	0·14^*^	0·40^**^	1·00	
Teaching nutrition (12)	− 0·12	− 0·13^*^	0·50^**^	0·11	− 0·05	0·42^**^	0·43^**^	0·25^**^	0·01	0·36^**^	0·26^**^	1·00

* *P* < 0.5.

** *P* < 0.01.

Table 6 shows that most of the subscales of the T-CFPQ showed significant correlations with each other. However, the highest correlations were found between encourage balance and variety, modelling and teaching nutrition (*r* = 0·53; 0·50, respectively) (*P* < 0·05). In other words, as the scores of modelling and teaching nutrition increase, the score of encourage balance and variety also increases.

The correlations of T-CFPQ subscale scores with the child’s BMI *z*-score and maternal BMI values were calculated, and the results are presented in Table 7.

**Table 7:** Correlation between Turkish version of the Comprehensive Feeding Practices Questionnaire subscales and child’s BMI z-score and mother’s BMI

	Child’s BMI *z*-score (*r*)	Mother’s BMI (*r*)
Child control	− 0·083	0·054
Emotion regulation	0·036	0·076
Encourage balance/variety	0·032	0·081
Healthy environment	0·014	0·032
Food as a reward	0·010	0·070
Involvement	0·008	− 0·121^*^
Modelling	− 0·063	− 0·067
Monitoring	− 0·049	− 0·050
Pressure to eat	− 0·173^**^	0·044
Restriction for health	0·061	− 0·020
Restriction for weight	0·339^**^	0·154^*^
Teaching nutrition	− 0·063	− 0·062

*Spearman correlation analysis.*

* *P* < 0.05.

** *P* < 0.01.

When Table 7 is examined, there is a negative, low-level statistically significant (*r* = –0·173; *P* < 0·01) correlation between the child’s BMI *z*-score and the pressure to eat, while there was a positive, moderate-level statistically significant (*r* = 0·339; *P* < 0·01) correlation between the child’s BMI *z*-score and restriction for weight. The higher the score for the pressure to eat, the lower the child’s BMI *z*-score value, while the higher the score for the restriction for weight, the higher the child’s BMI *z*-score value. A statistically significant negative correlation (*r* = –0·121; *P* < 0·05) was found between maternal BMI and the involvement dimension, while a statistically significant positive correlation (*r* = 0·154; *P* < 0·01) was found between maternal BMI and restriction for weight. Accordingly, when the involvement score increases, the mother’s BMI value decreases, and when the restriction for weight score increases, the mother’s BMI value increases.

## Discussion

This is the first study to test the reliability and validity of the T-CFPQ in a sample of Turkish parents with children aged 2–8 years. The study found that the version with twelve factors and forty-seven items was better adapted to Turkish culture than the original CFPQ, which consisted of forty-nine items and twelve factors^(15)^. In total, removing two items (items 18 and 42) was decided because their factor loadings were below 0·30. In the original CFPQ^(15)^, item 18 is related to ensuring that children do not consume too many fatty foods and is included under the restriction for weight. Shohaimi *et al.*^(20)^ evaluated the restriction items for weight control and reported that mothers were more restrictive about the consumption of high-fat foods (item 18) rather than the consumption of foods that may cause children to become obese, so the classification of item 18 as a restriction for weight control may be controversial. Foods that are high in fat contribute to overall fat and energy intake. Reducing the intake of high-fat foods that contain high levels of saturated fat, trans fat and cholesterol is important for maintaining good health and preventing chronic diseases^(31)^. However, the consumption of fatty foods alone is not enough to explain the development of obesity in children. Item 18 was excluded from our study as it did not explain the relevant factor sufficiently.

In the original CFPQ^(15)^, item 42 is a negative item about telling children what they should and should not consume without any explanation. It is included in the teaching nutrition and has a low factor loading. Similar to this study, Shohaimi *et al.* removed item 42 in their study^(20)^ because it disrupted the model fit. The low factor loading of this item was because of mothers encouraging their children to consume healthy and nutritious foods, but not fully explaining why they did not allow their children to consume any foods they wanted or why children could not consume these foods.

Shohaimi *et al.*^(20)^ conducted the study in Malaysia and confirmed the construct consisting of thirty-nine items and twelve factors. The psychometric properties of the CFPQ were assessed in a sample of Hispanic-American preschool children aged 2–5 years (*n* 187). Thirty-four items and a five-factor structure consisting of monitoring, restriction for weight, promotion of overconsumption, healthy eating guidance and healthy eating variety were found to be supported^(32)^. In studies in other countries such as France^(21)^, Norway^(33)^, Brazil^(34)^, Iran^(35)^ and New Zealand^(18)^, the original CFPQ structure was not supported. The reason for the presence of different constructs in these studies was associated with ethnicity, differences in socio-economic status and the examination of different age groups.

Encourage balance/variety was reported to have low internal consistency both in the original CFPQ^(15)^ and in the study by Shohaimi *et al.*^(20)^, but the lowest Cronbach’s alpha value in our study was the healthy environment (α = 0·58). Similar to other studies, the Cronbach’s alpha value of involvement was found to be below 0·60^(15,20)^. Similar to the original CFPQ, the Cronbach’s alpha value of monitoring is consistent with studies conducted both in Turkish culture and in other countries^(17–20,35)^. This suggests that the monitoring and related items are understood well by different cultures and that it is a valid construct.

In their study, Melbye *et al.*^(33)^ found a correlation between encourage balance/variety and teaching nutrition. Encourage balance/variety and teaching nutrition are related to communicating with the child about nutrition. In our study, as the scores of the encourage balance/variety increased, the scores of the supporting communication with the child about nutrition such as teaching nutrition, healthy environment, involvement, modelling and monitoring increased. Moreover, healthy eating practices (modelling) demonstrated by parents are closely related to the creation of a healthy food environment at home. A study reported that parents often interpret applying eating pressure to their children as a simple show of affection and positive behaviour^(32)^. In the original CFPQ, the authors stated that the use of food as a reward and eating pressure were interrelated concepts^(15)^. Similarly, this study found that pressure to eat increased with higher scores for food as a reward.

Parental concern regarding their child’s body weight is associated with high levels of restraint and the child’s body weight^(36)^. It is important to distinguish the reasons for parental restraint, as it can be influenced by various factors such as the child’s health conditions, weight management, teaching healthy eating habits for the future, religious beliefs and more^(33)^. In the original scale, restriction for health and restriction for weight are examined in two different dimensions, and this study supports the original CFPQ^(15)^. In the original study, parents with overweight children were reported to restrict for both health and weight control, whereas parents with underweight children showed less restriction for weight control and more eating pressure. The study found that children who were subjected to restriction for weight had a higher BMI *z*-score, while those who experienced more eating pressure had a lower BMI *z*-score. Mothers with overweight/obese children may put less pressure on their children to eat because they are more concerned about their children’s body weight. In addition, as children’s body weight increases, mothers may show restrictive feeding behaviours, and their feeding practices may change according to their children’s body weight. Moreover, mothers with higher restrictions for weight control scores also had higher BMI.

### Limitations and future directions

Parents’ dietary practices are affected by income status, and parents may be forced to turn to unhealthy food intake even if they do not want to because it may be affordable. Education level as well as income level affected the dietary practices of the parents. In this study, the fact that the income level of most of the parents was medium and the education level of the mothers was high may be a limitation of the use of the snowball sampling method. It is recommended to repeat the study in larger samples with a balanced distribution of income and education levels. In addition, the fact that anthropometric measurements such as body weight and height of the children were taken according to the statements of the parents is an important limitation of the study. Although the mother is often the person responsible for child nutrition in our country, it is known that the approaches of both parents have an impact on the child. As the study sample only includes mothers, it cannot be considered representative of the approach of both parents (mother and father). Therefore, it is recommended that future studies include both parents.

## Conclusion

In this study, we found that the psychometric properties of the adapted T-CFPQ were similar to the original CFPQ except for two items and that the T-CFPQ, with minor modifications, was a valid instrument for assessing parental feeding practices in a sample of Turkish parents with children aged 2–8 years. The study focused on mothers of preschool children because the preschool period is a developmental period of increased autonomy and exposure to new foods. In this process, parents play a major role in the development of healthy eating habits and correct behaviours related to nutrition to prevent excessive body weight gain in children. Although this scale was developed as a research tool, it will provide a different perspective to health professionals working with overweight or obese children by revealing the child and family interaction on nutrition through various factors. It can also be used as an evaluation tool for educational programmes aiming to improve the nutritional relationship between children and parents. The results of the study suggest that the T-CFPQ is a promising tool for future studies in assessing the information needed about parent-child nutritional interactions.

## Supporting information

Şarahman-Kahraman et al. supplementary material Şarahman-Kahraman et al. supplementary material

## Supplementary material

For supplementary material accompanying this paper, visit https://doi.org/10.1017/S1368980024001125.
