# Supplementary material for: Investigation of the psychometric properties of the Comprehensive Feeding Practices Questionnaire in Turkish parents
Source: Public Health Nutr. 2024 May 30;27(1):e148. doi: 10.1017/S1368980024001125 (PMC11617419; doi:10.1017/S1368980024001125)
Supplement: Şarahman-Kahraman et al. supplementary material [file S1368980024001125sup001.docx]

**Supplementary Material**

**Monitoring (MN) / İzleme**

**Item 1.** How much do you monitor the sweets (candy, ice cream, cakes, pies, pastries) your child consumes?

Çocuğunuzun tükettiği tatlıları (şeker, dondurma, kek, turta, hamur işleri) ne kadar takip ediyorsunuz?

**Item 2.** How much do you monitor the types of junk food (potato chips) your child consumes?

Çocuğunuzun tükettiği abur cuburları (cips çeşitleri) ne kadar takip ediyorsunuz?

**Item 3.** How much do you monitor the high-fat foods your child consumes?

Çocuğunuzun tükettiği yüksek yağlı yiyecekleri ne kadar takip ediyorsunuz?

**Item 4.** How much do you monitor the sugary drinks your child consumes (fruit soda, oralet, etc.)?

Çocuğunuzun tükettiği şekerli içecekleri (meyveli gazoz, meyveli soda, oralet vb.) ne kadar takip ediyorsunuz?

**Child control (CC) / Çocuk kontrolü**

**Item 5.** Do you let your child eat what they want?

Çocuğunuzun istediğini yemesine izin veriyor musunuz?

**Item 6.** At dinner, do you let your child choose what he/she wants from the food offered?

Akşam yemeğinde, çocuğunuza sunulan yiyeceklerden istediğini seçmesine izin veriyor musunuz?

**Item 10.** If your child doesn't like the food you serve them, do you prepare something else?

Çocuğunuz ona sunduğunuz yemeği beğenmezse başka bir şey hazırlar mısınız?

**Item 11.** Do you allow your child to eat snacks when they feel like it?

Çocuğunuzun canı istediğinde atıştırmalıkları tüketmesine izin veriyor musunuz?

**Item 12.** Do you allow your child to leave the table when he/she is full, even if others in the family have not finished eating?

Ailedeki diğer bireylerin yemekleri bitmemiş olsa bile, çocuğunuz doyduğunda sofradan kalkmasına izin veriyor musunuz?

**Emotion regulation (ER) / Duygu düzenleme**

**Item 7.** When your child is cranky, is the first thing you do is give them something to eat or drink?

Çocuğunuz huysuzlaştığında ilk yaptığınız şey ona yiyecek veya içecek bir şeyler vermek midir?

**Item 8.** Do you give your child something to eat or drink when you think they are not hungry but they are bored?

Çocuğunuzun aç olmadığını düşündüğünüz halde canı sıkıldığında yemesi veya içmesi için bir şeyler veriyor musunuz?

**Item 9.** Do you give your child something to eat or drink if they are upset but you think they are not hungry?

Çocuğunuzun aç olmadığını düşündüğünüz halde üzgünse yemesi veya içmesi için bir şeyler veriyor musunuz?

**Encourage balance and variety (EB) / Dengeyi ve çeşitliliği teşvik etme**

**Item 13.** Do you encourage your child to eat healthy foods before unhealthy foods?

Çocuğunuzu sağlıksız yiyeceklerden önce sağlıklı yiyecekler tüketmeye teşvik ediyor musunuz?

**Item 24.** I encourage my child to try new foods.

Çocuğumu yeni besinler denemeye teşvik ederim.

**Item 26.** I tell my child that healthy food is delicious.

Çocuğuma sağlıklı besinlerin lezzetli olduğunu söylerim.

**Item 38.** I encourage my child to eat a variety of foods.

Çocuğumu çeşitli yiyecekler tüketmeye teşvik ederim.

**Healthy environment (HE) / Sağlıklı çevre**

**Item 14.** Most of the food I keep at home is healthy.

Evde bulundurduğum yiyeceklerin çoğu sağlıklıdır.

**Item 16.** I keep a lot of junk food (chips) in my house. **R***

Evimde çok fazla abur cubur (cips çeşitleri) bulundururum.

**Item 22.** My child is offered healthy food choices at every meal.

Çocuğuma her öğünde sağlıklı yiyecek seçenekleri sunulur.

**Item 37.** I keep a lot of sweets (candy, ice cream, cakes, pies, pastries) in my house. **R***

Evimde çok fazla tatlı (şeker, dondurma, kek, turta, hamur işleri) bulundururum.

**Involvement (IN) / Katılım**

**Item 15.** I involve my child in planning family meals.

Çocuğumu aile yemeklerinin planlanmasına dahil ederim.

**Item 20.** I let my child help prepare family meals.

Çocuğumun aile yemekleri hazırlanırken yardım etmesine izin veririm.

**Item 32.** I encourage my child to participate in grocery shopping.

Çocuğumu market alışverişine katılmaya teşvik ederim.

**Pressure to eat (PE) / Yeme baskısı**

**Item 17.** My child should always eat all the food on their plate.

Çocuğum her zaman tabağındaki bütün yiyecekleri tüketmelidir.

**Item 30.** Even if my child says, "I'm not hungry," I still try to get them to eat something.

Çocuğum “aç değilim” dese bile, yine de ona bir şeyler yedirmeye çalışırım.

**Item 39.** If my child eats little, I try to get him/her to eat more.

Çocuğum az yemek yerse daha fazla yemesi için çaba gösteririm.

**Item 49.** When he/she says he/she has finished eating, I try to get him/her to eat one/two more bites of food.

Yemeğini bitirdiğini söylediğinde, çocuğuma bir/ iki lokma daha yemek yedirmeye çalışırım.

**Food as a reward (FR) / Ödül olarak yiyecek**

**Item 19.** I offer my child his or her favorite foods in exchange for good behavior.

Çocuğuma iyi davranışları karşılığında en sevdiği yiyecekleri sunuyorum.

**Item 23.** I give my child sweets (candy, ice cream, cake, pastries) as a reward for good behavior.

Çocuğuma iyi davranışlarının ödülü olarak tatlı (şeker, dondurma, kek, hamur işleri) veririm.

**Item 36.** I forbid my child from consuming sweets/sweets as a reaction to bad behavior.

Kötü davranışlarına tepki olarak çocuğumun şekerleme/tatlı tüketmesini yasaklıyorum.

**Restriction for health (RH) / Sağlık için kısıtlama**

**Item 21.** If I didn't guide or regulate my child's food choices, he would consume too much of the foods he likes.

Çocuğumun besin tercihlerine rehberlik etmeseydim veya düzenlemeseydim, sevdiği yiyecekleri çok fazla tüketecekti.

**Item 28.** If I didn't guide or regulate my child's food choices, he would eat too much junk food.

Çocuğumun besin tercihlerine rehberlik etmeseydim veya düzenlemeseydim, çok fazla abur cubur tüketecekti.

**Item 40.** I have to make sure that my child doesn't eat too much of the foods he or she likes.

Çocuğumun sevdiği yiyecekleri çok fazla tüketmediğinden emin olmalıyım.

**Item 43.** I have to make sure that my child does not eat too many sweets (candy, ice cream, cakes or pastries).

Çocuğumun çok fazla tatlı (şeker, dondurma, kek veya hamur işleri) tüketmediğinden emin olmalıyım.

**Teaching nutrition (TN) / Beslenme hakkında eğitim**

**Item 25.** I discuss with my child why it is important to eat healthy foods.

Çocuğumla sağlıklı besinleri tüketmenin neden önemli olduğunu tartışırım.

**Item 31.** I discuss the nutritional value of food with my child.

Çocuğumla yiyeceklerin besin değerini tartışırım.

**Restriction for weight (RW) / Ağırlık kontrolü için kısıtlama**

**Item 27.** I encourage my child to eat less so that she doesn't get fat.

Şişmanlamasın diye çocuğumu daha az yemeye teşvik ederim.

**Item 29.** I give my child small portions at meals so that he/she can control his/her body weight.

Vücut ağırlığını kontrol edebilmesi için çocuğuma öğünlerde küçük porsiyonlar veririm.

**Item 33.** If my child eats more than usual at one meal, I try to limit what they eat at the next meal.

Çocuğum bir öğünde normalden fazla yerse, bir sonraki öğünde yediklerini kısıtlamaya çalışırım.

**Item 34.** I restrict the foods my child consumes that can make him/her fat.

Çocuğumun tükettiği ve onu şişmanlatabilecek yiyecekleri kısıtlarım.

**Item 35.** There are some foods that my child should not eat because they can make them fat.

Şişmanlatacağı için çocuğumun tüketmemesi gereken bazı yiyecekler vardır.

**Item 41.** I don't want my child to get fat, so I don't allow him to eat between meals.

Çocuğumun şişmanlamasını istemediğim için öğün aralarında bir şeyler tüketmesine izin vermiyorum.

**Item 45.** I often put my child on a diet to control their body weight.

Çocuğumun vücut ağırlığını kontrol etmek için sık sık diyet uygulatırım.

**Modeling (MD) / Modelleme**

**Item 44.** I try to set an example for my child about healthy eating by consuming healthy foods.

Sağlıklı besinler tüketerek çocuğuma sağlıklı beslenme konusunda örnek olmaya çalışıyorum.

**Item 46.** I try to eat healthy foods in front of my child, even if they are not my favorite foods.

Sevdiğim yiyecekler olmasa bile çocuğumun önünde sağlıklı besinler tüketmeye çalışırım.

**Item 47.** I try to appear enthusiastic about eating healthy foods.

Sağlıklı besinler tüketme konusunda istekli görünmeye çalışırım.

**Item 48.** I show my child how much I enjoy eating healthy food.

Çocuğuma sağlıklı besinleri tüketmekten ne kadar keyif aldığımı gösteriyorum.

**Note:** Appendix includes all items retained in final scale. Items 18 and 42 were excluded from the analysis.

**Item 18.** I have to be sure that my child does not eat too many high-fat foods. (RW factor)

Çocuğumun yağlı yiyecekleri çok fazla tüketmediğinden emin olmalıyım.

**Item 42.** I tell my child what to eat and what not to eat without explanation. (TN factor)

Açıklama yapmadan çocuğuma ne yiyip ne yememesi gerektiğini söylerim.

Items numbered 1–13 utilize a 5-point response scale “never, rarely, sometimes, mostly, always” Items numbered 14–49 utilize a 5-point scale with different, “disagree, slightly disagree, neutral, slightly agree, agree” Items with R are reverse coded.
